# 

**Published:** 1894-10-20

**Authors:** 


### CONTENTS.

**The Chelsea Hospital for Women** - - - - - - - 37

**Appendicitis** - - - - - - - - - - - 38

**On Modern Progress in Ophthalmic Medicine and Surgery.**—By R. BRUDENELL CARTER.—Inflammation of the Iris - - - - - - - - - - - 39

**The First Century of English Medical Literature.**—IX. Some Later Herbalists - - - - - - - - - 40

**Notes and News** - - - - - - - - - - - 42

**Medical Progress and Hospital Clinics:**

Ventro-Fixation of the Uterus - - - - - - - 43

Case of Hysterical Knee- - - - - - - - - 44

PROGRESS IN MEDICINE.—Infective Diseases - - - - 46

NEW APPLIANCES AND THINGS MEDICAL - - - - - - 48

**Annotations.**—Mr. Bryant on the Public and the Profession—Sir James Paget on the Scientific Temper—The Clinical Society - 41

**The Institutional Workshop:**

HOSPITAL CONSTRUCTION.—The New Institution for Epileptics in Berlin - - - - - - - - - - - 49

PRACTICAL DEPARTMENTS—Water Pressure Accumulator - - 50

HOSPITAL FESTIVALS AND MEETINGS - - - - - - - 50

PRACTICAL POINTS - - - - - - - - - - 51

THE STORY OF THE INSANE FROM YEAR TO YEAR - - - 51

CONSTRUCTION NOTES - - - - - - - - - - 52

**Scraps and Gleanings** - - - - - - - - - 52

**The Book World of Medicine and Science** - - - - 53

**The Student of Medicine** - - - - - - - - 53

### EXTRA SUPPLEMENT.—THE NURSING MIRROR.

**News from the Nursing World.**—Nursing at Guy's Hospital—The Royal Free Hospital—Over the Hospital Wall—District Nursing at Stockport—A Useful Institution—Sick Paupers in the New Forest Union—Is Nursing Profitable?—Aberdeen—Nurses in France—Nurses in Ceylon—Disappointed Burglars—Training for Male Nurses in U.S.A.—Nurses at Boston - xvii
**Nurses as Sanitarians** - - - - - - - - - xix
**Nursing in Rio de Janiero—Our American Letter** - xx
**Notes for Nurses on Antiseptic Surgery.**—By WILLIAM HORROCKS, M.D., F.R.C.S.—II. Treatment of the Psoas Abscess - - - - - - - - - - - - xix
**Appointments** - - - - - - - - - - - - xx
**Minor Appointments** - - - - - - - - - - xx
**Everybody's Opinion** - - - - - - - - - - xxi
**Nurses at the Addenbrooke Hospital** - - - - - xxi
**Dress and Uniforms.**—III. - - - - - - - - xxii

